# Mapping of integrated PIN diodes with a 3D architecture by scanning microwave impedance microscopy and dynamic spectroscopy

**DOI:** 10.3762/bjnano.11.159

**Published:** 2020-11-23

**Authors:** Rosine Coq Germanicus, Peter De Wolf, Florent Lallemand, Catherine Bunel, Serge Bardy, Hugues Murray, Ulrike Lüders

**Affiliations:** 1 Normandie Université, ENSICAEN, UNICAEN, CNRS, CRISMAT, 14000 Caen, France; 2 Bruker Nano Surfaces, 112 Robin Hill Road, CA 93117, Santa Barbara, USA; 3 Murata Integrated Passive Solutions, 2 Rue de la Girafe, 14000 Caen, France; 4 NXP Semiconductors, Esplanade Anton Philips 2, 14905, Colombelles, France

**Keywords:** atomic force microscopy (AFM), DataCube, doping, integrated PIN diode, nanoprobing, scanning probe microscopy (SPM), scanning microwave impedance microscopy (sMIM), spectroscopy

## Abstract

This work addresses the need for a comprehensive methodology for nanoscale electrical testing dedicated to the analysis of both “front end of line” (FEOL) (doped semiconducting layers) and “back end of line” (BEOL) layers (metallization, trench dielectric, and isolation) of highly integrated microelectronic devices. Based on atomic force microscopy, an electromagnetically shielded and electrically conductive tip is used in scanning microwave impedance microscopy (sMIM). sMIM allows for the characterization of the local electrical properties through the analysis of the microwave impedance of the metal–insulator–semiconductor nanocapacitor (nano-MIS capacitor) that is formed by tip and sample. A highly integrated monolithic silicon PIN diode with a 3D architecture is analysed. sMIM measurements of the different layers of the PIN diode are presented and discussed in terms of detection mechanism, sensitivity, and precision. In the second part, supported by analytic calculations of the equivalent nano-MIS capacitor, a new multidimensional approach, including a complete parametric investigation, is performed with a dynamic spectroscopy method. The results emphasize the strong impact, in terms of distinction and location, of the applied bias on the local sMIM measurements for both FEOL and BEOL layers.

## Introduction

In “front end of line” (FEOL) processing, the control, detection, and quantification of the effective 2D distributions of active dopants in semiconductors are crucial to optimize and increase the device integration. In order to map the electrical properties of microelectronic materials with a high spatial resolution, scanning probe microscopy (SPM), based on atomic force microscopy (AFM), offers several modes based on the control of electrical conduction and on the measurements of the electrical interactions of a biased tip–sample nanoscale system [1–2]. Scanning spreading resistance microscopy (SSRM) and scanning capacitance microscopy (SCM) [3–5] are modes widely used for the detection of charge carriers, carrier types, and density of defects. These modes provide sub-10 nm two-dimensional maps of the electrical properties of doped semiconductor layers [1,6–8] when a fixed bias is applied to the nanoscale contact.

In SSRM, a DC voltage is applied to the sample and the resulting current, flowing from the conductive tip through the sample to the back contact, is recorded using a logarithmic amplifier with a wide dynamic range [9–10]. Based on the measured current, the overall equivalent resistance, including the conductive tip resistance, the spreading resistance of the semiconductor under the contact, the bulk resistance of the sample, and the back-contact resistance [11], is experimentally determined. Therefore, at a fixed applied bias, the SSRM measurements map the variation in concentration of mobile majority carriers in doped semiconductors. A high load on the tip is required to obtain the spreading resistance. In fact, for silicon, a body-centred tetragonal configuration (β-Sn, also called Si-II) must be formed from the initial cubic diamond silicon (Si-I) in the highly stressed region just below the tip [12]. Furthermore, in air and under ambient conditions, due to the interaction between the tip and the surface, the probe can dig into the sample and cause material removal during the scan. This nanomechanical behaviour can be exploited for 3D-SSRM [13–15], but it can also induce irreversible surface modifications of the semiconductor [11].

Scanning capacitance microscopy (SCM) is applied to characterize the majority carrier concentration and carrier types in semiconductors. In SCM, an electrically conductive tip scans in contact with the analysed sample surface. The tip and the probed sample volume, during contact, represent a metal–insulator–semiconductor (MIS) structure at the nanoscale [4,16–17]. A low-frequency (approx. 100 kHz) AC voltage (*V*_AC_) is applied to the tip–sample system in order to generate the movement of free carriers [18]. At a fixed DC bias, the capacitance variation is measured with a highly sensitive capacitance sensor with a sensitivity of approx. 10^−19^ F·Hz^−1/2^. In addition, the detected phase sign indicates the majority carrier type and, hereby, enables the distinction between n-type and p-type semiconductor regions. In this way, SCM is applied to map the local carrier concentration and carrier type in semiconductor materials with a sensitivity between 10^15^ and 10^20^ atoms·cm^−3^ and with the spatial resolution of AFM [17,19].

However, there are limits to the SCM and SSRM techniques. For example, for SCM, when the local carrier concentration is extremely low, the SCM signal may be undetectable and close to zero. For SSRM, a very good control of the applied force is necessary to record the spreading resistance. However, depending on the local properties of the material, the optimum force can vary in a same scan.

More recently, scanning microwave impedance microscopy (sMIM) [20–21] was implemented on AFM. The originality of this mode comes from the use of an electromagnetically shielded and electrically conductive tip [20]. The sMIM mode is based on the combination of the AFM capabilities, in terms of imaging with a high spatial resolution and versatility, and the near-field interaction of an incident microwave with the studied material. From the reflected signal, the local electrical properties of the scanned material are determined without the need of an applied voltage. Some studies reported sMIM measurements performed in doped semiconductors [22] deposited onto FEOL layers, ferroelectrics [23], or 2D materials [22,24].

Here, sMIM was employed to analyse a monolithic silicon integrated PIN diode with a 3D architecture. The studied device includes FEOL regions (doped semiconductors and oxides) and “back end of line” (BEOL) regions (metallic and dielectric layers). After the presentation of the results, the capabilities of the sMIM mode are discussed in detail. In addition, a high-resolution dynamic parametric study based on the acquisition of sMIM signals as a function of the applied *V*_DC_ bias, at each pixel, is carried out.

## Experimental sMIM Measurements

**Figure 1:**
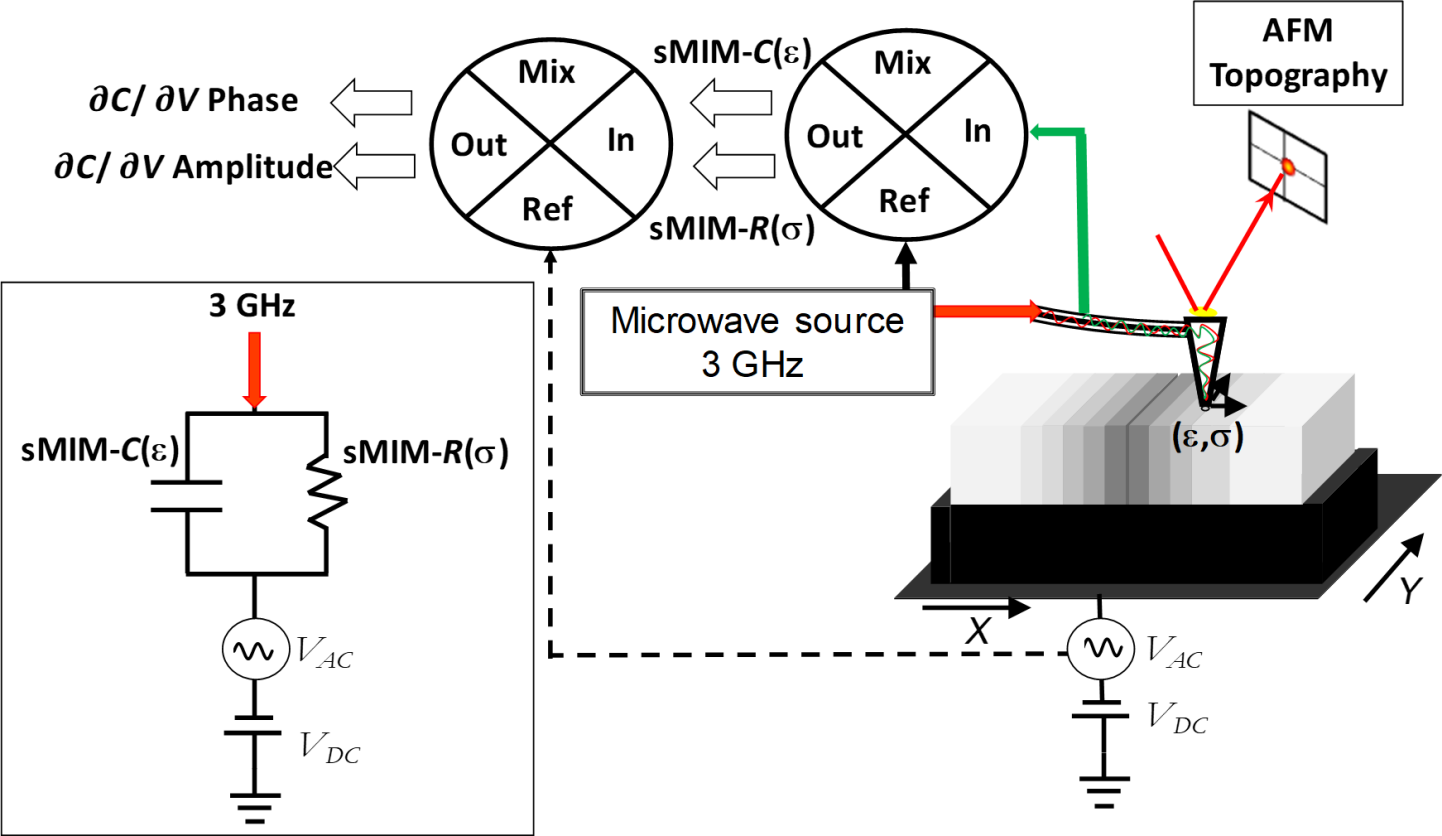
sMIM setup showing the detection of the sMIM-*C* (ε) and sMIM-*R* (σ) signals. *V*_AC_ and *V*_DC_ are applied to detect ∂*C*/∂*V* signals. Box: equivalent circuit of the RF nanoscale contact.

Based on AFM, the sMIM mode is a microwave mode in which an incident microwave signal travels through an electromagnetically shielded and electrically conductive tip [21]. This special tip enhances the microwave sensitivity and eliminates parasitic noise. The tip design and its properties are described elsewhere [25]. A gigahertz microwave signal travels through the tip and interacts with a local volume of the sample material under the tip. Optimized for an incident frequency of approx. 3 GHz, the subsurface analysis volume extends to a depth of approx. 100 nm. For a material with a conductivity σ and a permittivity ε, the AFM sMIM tip–sample impedance is a complex impedance [26], equivalent to a capacitor in parallel with a resistance (Figure 1). The incident microwave signal interacts with the sample resulting in a transmitted wave and a reflected wave. Since the probe is shielded, the stray capacitance formed by the cantilever and the sample can be neglected. After a calibration step and amplification and demodulation of the reflected signal (Figure 1), the complex admittance of the tip–sample impedance is given by the real part of sMIM (sMIM-*R*) and by the imaginary part of sMIM (sMIM-*C*) [27–28]. With a second quadrature mixer (Figure 1), the ∂*C*/∂*V* amplitude and ∂*C*/∂*V* phase are also measured at each pixel.

In this study, the sMIM measurements were performed using a Bruker Dimension Icon AFM, equipped with a Primenano Scanwave (except the data in Figure 9, which was acquired using a Primenano Scanwave Pro), and sMIM tips with a radius of approx. 50 nm. A scan rate of 1 Hz was employed and 512 pixels per line were measured. It is interesting to note that, unlike the SCM and SSRM methods, no bias voltage is required to perform sMIM imaging. Furthermore, in order to record ∂*C*/∂*V* signals and to compare them to sMIM measurements, imaging was performed with a *V*_AC_ bias of 1 V at a frequency 90 kHz and *V*_DC_ = 0 V. The signals of ∂*C*/∂*V* phase, ∂*C*/∂*V* amplitude, sMIM-*R*, sMIM-*C*, and the topography were simultaneously acquired. In a second investigation, dynamic sMIM spectroscopy was performed by sweeping the *V*_DC_ voltage from −2.0 V to 2.5 V at each location of the scan.

## The Integrated PIN Diode Device

For the design of RF switches, we fabricated a highly integrated monolithic PIN diode with a 3D architecture. In the context of downscaling technology, the PIN diode is co-designed with a vertical structure inside the wafer. For RF applications, highly integrated and high-quality passive components, such as 3D capacitors, precision resistors, and inductors are added, designed, and fabricated into the same wafer [29–30]. The integration of PIN diodes into conventional planar IC processes has not been very popular because such elements need to coexist with other devices in the chip and the diode components need to be electrically isolated. In our case, in order to electrically isolate the devices from one another, deep trenches were integrated in the process. For RF switching applications, a circular topology was chosen to avoid any right-angled corners that may induce edge effects in RF.

The starting material is a p-type Si substrate with relatively high ohmic resistance (1 kΩ·cm), oriented along the ⟨100⟩ direction. The first step in the process consists of forming an n-type doped buried layer (BN) on the top of the wafer (through implantation and diffusion of the doping species). A lightly doped n-type epitaxial silicon layer is, then, grown on top of the buried layer using chemical vapour deposition. The targeted epitaxial layer thickness is 7.5 µm with a resistivity of 12 Ω·cm. The anode of the diode is formed by a 30 µm diameter p^+^ layer, also made by the implantation and diffusion of electron-acceptor species. Therefore, the intrinsic layer, acting as an insulator due to its low doping concentration, is stacked in between the p-type and n-type layers. The studied sample came from a fully processed wafer, designed for power electronic applications, in which both FEOL and BEOL steps were accomplished.

The SPM electrical measurements were performed in the cross section of the chip at the wafer level. In order to enable a stable and constant nanoscale contact between the sensor tip and the sample, a surface with a low roughness is required. For this purpose, the sample was hand-polished down to a roughness of a few nanometres with diamond-based lapping films with decreasing granularity. In the following section, the local electrical properties of all layers in the cross section of the PIN diode are analysed. In order to evaluate the impact of the applied *V*_DC_ bias, an electrical back contact is created between the microscope chuck and the sample.

## Results and Discussion

### The vertical PIN structure

**Figure 2:**
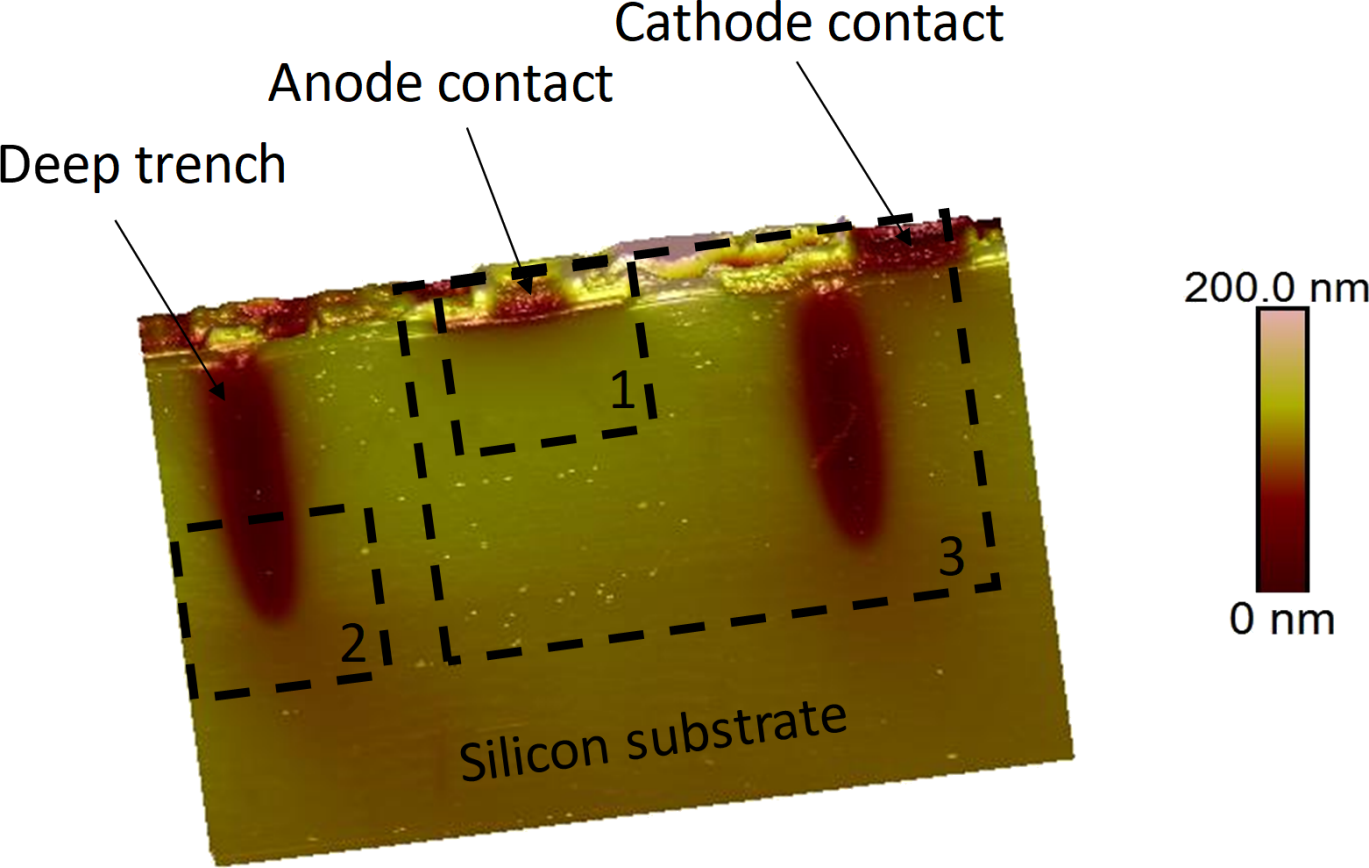
Topography of the cross section of the PIN diode. The scanned area is 83 µm × 54 µm. Deep trenches and metallic contacts are localized and indicated. The three dashed black boxes indicate the location of the subsequent sMIM scans.

Figure 2 shows the surface topography of the cross section of the PIN diode. The different materials used (silicon substrate, epitaxial layers, oxides, and alloy metals) have a slightly different polishing rate, which results in the observed topography. In the AFM topography image, one can localize the two deep trench isolation structures in the silicon wafer, as well as the anode and cathode contacts. It is important to note that a low roughness is required for a stable tip–sample contact during the sMIM measurements. A root mean square (RMS) roughness of the silicon surface below 3 nm was measured.

**Figure 3:**
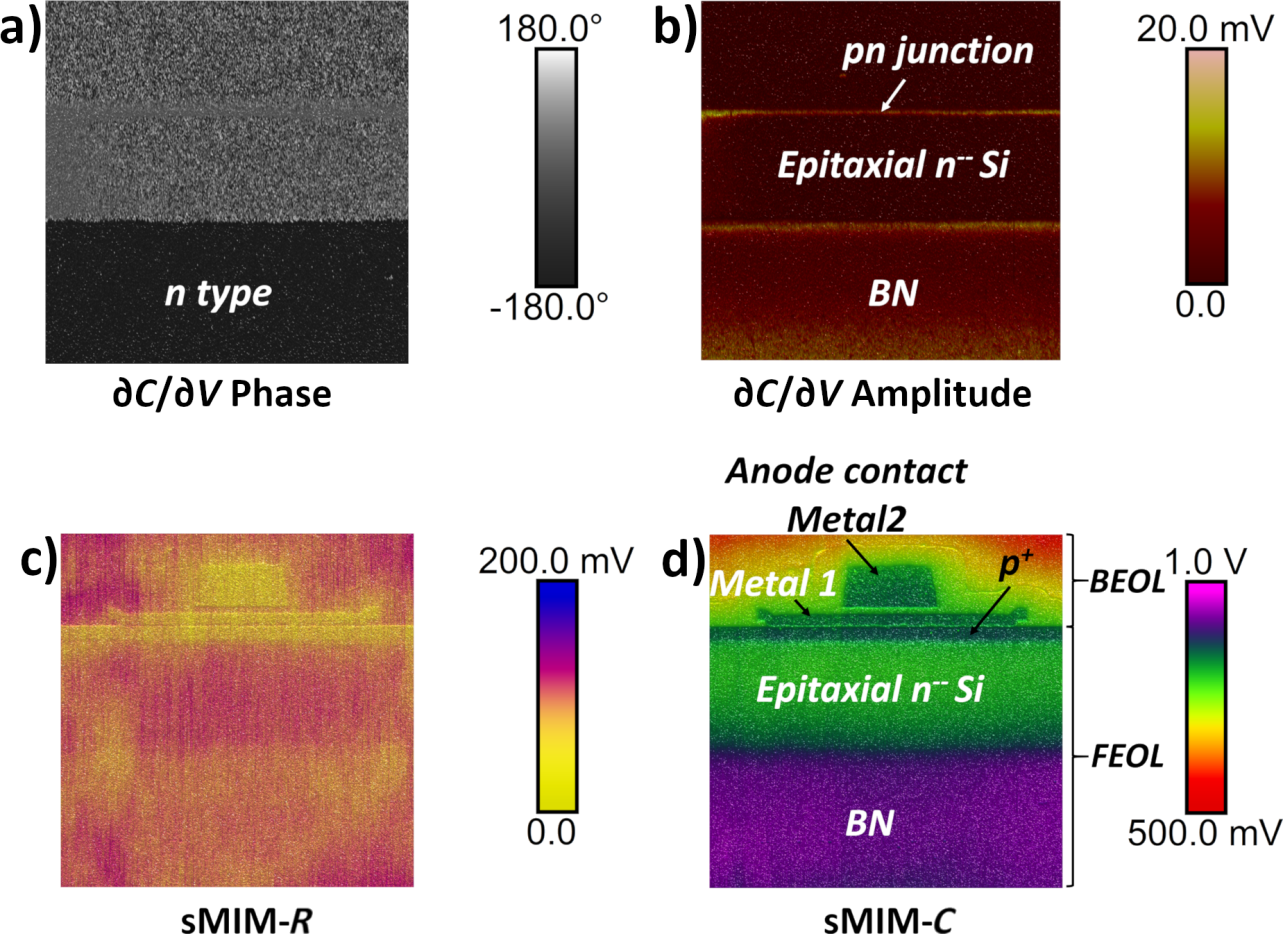
sMIM results for a 25 µm × 25 µm area at the PIN anode area (area 1 in Figure 2). (a) ∂*C*/∂*V* phase, (b) ∂*C*/∂*V* amplitude, (c) sMIM-*R*, and (d) sMIM-*C*. All FEOL and BEOL layers can be observed.

First, we studied the PIN vertical structure and the anode contact. Area 1 in Figure 2 was scanned with *V*_DC_ = 0 V and *V*_AC_ = 1.0 V. The sMIM results for a scanned area of 25 µm × 25 µm, centred on the anode contact, are presented in Figure 3. All four measured properties: ∂*C*/∂*V* phase, ∂*C*/∂*V* amplitude, sMIM-*R*, and sMIM-*C* (recorded simultaneously with the topography) are displayed. The ∂*C*/∂*V* phase and ∂*C*/∂*V* amplitude data (Figure 3a and Figure 3b, respectively) are sensitive to the variation in the majority carrier concentration in the FEOL layers of the device. They allow one to clearly distinguish between the p^+^ anode implanted layer and the buried n-type layers of the diode. In the ∂*C*/∂*V* amplitude map (Figure 3b) we can observe two bright coloured lines in which the values of the ∂*C*/∂*V* amplitude are the strongest. The two signals localize the electrical junctions of the diode. The first signal is the signature of the pn junction, between the p^+^ implanted layer (anode) and the lightly doped n-type epitaxial silicon layer, and the second signal is the quasi-intrinsic/BN junction. Moreover, the amplitude of the local capacitance variation for the lightly doped n-type epitaxial silicon layer is close to zero (almost no signal). In the phase measurement, this layer has a value that is similar to the one obtained for the oxide, which explains the relatively poor contrast in the scan. The mappings of the equivalent resistance (i.e., sMIM-*R*) and capacitance (i.e., sMIM-*C*) of the nanoprobing system, during interaction with the incident 3 GHz microwave, are represented in Figure 3c and Figure 3d, respectively. These sMIM signals reveal the complete structure of the vertical PIN diode with the associated metallic contacts of the anode. Although the sMIM-*R* signal shows a low signal-to-noise ratio, it is still possible to differentiate metallic materials from semiconductor layers. Also, the conductivity continuity between the metallic contact and the p^+^ anode can be seen. The sMIM-*C* map clearly shows the complete architecture of the PIN structure with details of the anode contact and with very clear differences (in terms of contrast, layer location, and signal-to-noise ratio) between the P (p^+^ anode), I (lightly doped n-type epitaxial silicon), and N (n-type doped buried layer) layers.

**Figure 4:**
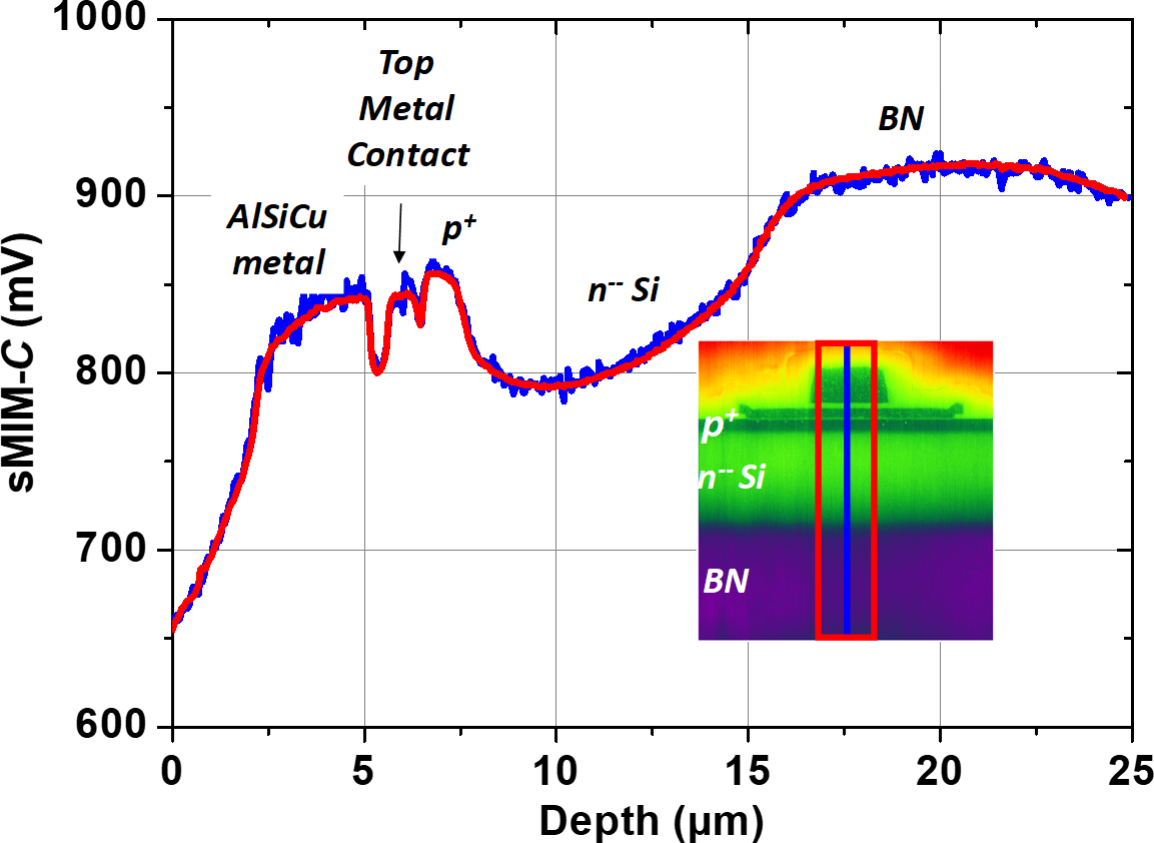
sMIM-*C* profiles along the central axis of the diode structure. A single line scan (blue) and the average of 100 line scans (red) are both represented. The corresponding regions are drawn in the inset of the sMIM-*C* mapping.

In order to check the reproducibility of the acquired sMIM-*C* dataset, cross-sectional profiles of the data along the central axis of the contact are presented in Figure 4. The results of a single line are compared with the average profile calculated for 100 line scans. They show that the depth profile has a good signal-to-noise ratio for the sMIM-*C* signal for each layer.

This first analysis demonstrates the capability of the sMIM methodology to distinguish, localize, and determine both p-type and n-type semiconductors as well as lightly doped layers, metallic contacts, and oxides with a good reproducibility and stability during the scan. The thickness of the different layers in the diode structure (determined for *V*_DC_ = 0 V and *V*_AC_ = 1.0 V) can be extracted from the sMIM-*C* profile. The p^+^ (anode) layer has a thickness of approx. 780 nm, whereas the lightly doped n-type epitaxial silicon layer has a thickness of approx. 7.5 µm. In this high-integration process, the control of the lightly doped epitaxial layer (also called intrinsic layer) in terms of spatial configuration (thickness) and local electrical properties of the PIN structure is a key parameter. In fact, this intrinsic layer allows one to meet the required electrical specifications for switches in power electronics.

**Figure 5:**
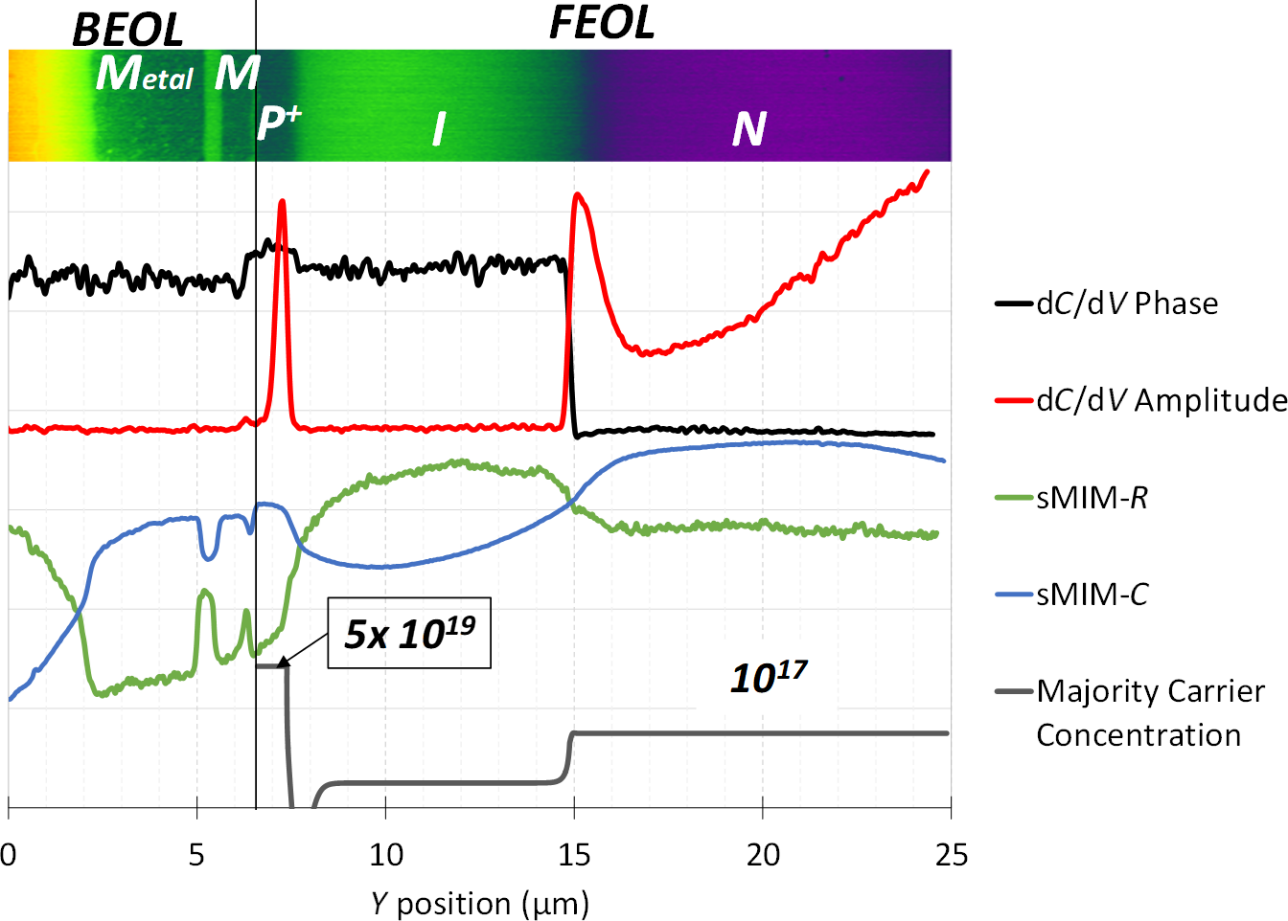
Depth profiles (∂*C*/∂*V* phase, ∂*C*/∂*V* amplitude, sMIM-*R*, and sMIM-*C*) along the PIN structure compared with simulated concentration values for the majority carriers.

In order to analyse the profiles extracted from the sMIM measurements, the majority carrier concentration calculated by numerical simulations [31] for an abrupt PIN doping profile is compared with the measured sMIM-*C* profile. We can see that there is a good correlation between the SMIM-*C* profile and the simulated majority carrier concentration profile. (Figure 5), and the two junctions P/I and I/N are well defined by the experimental data. In sMIM-*C*, there is a monotonic relationship between the observable (capacitance) and the material property of interest (carrier concentration), while the ∂*C*/∂*V* amplitude shows a non-monotonic relationship with the carrier concentration. This makes the interpretation and quantification of the sMIM-*C* data more straightforward in comparison to the ∂*C*/∂*V* amplitude data. For a better comprehension, see Supporting Information File 1, in which sMIM-*C* data and ∂*C*/∂*V* amplitude data are detailed for the spectra collected on a staircase reference sample with different known carrier concentration values for n- and p-type layers.

### Deep trench isolation structures

**Figure 6:**
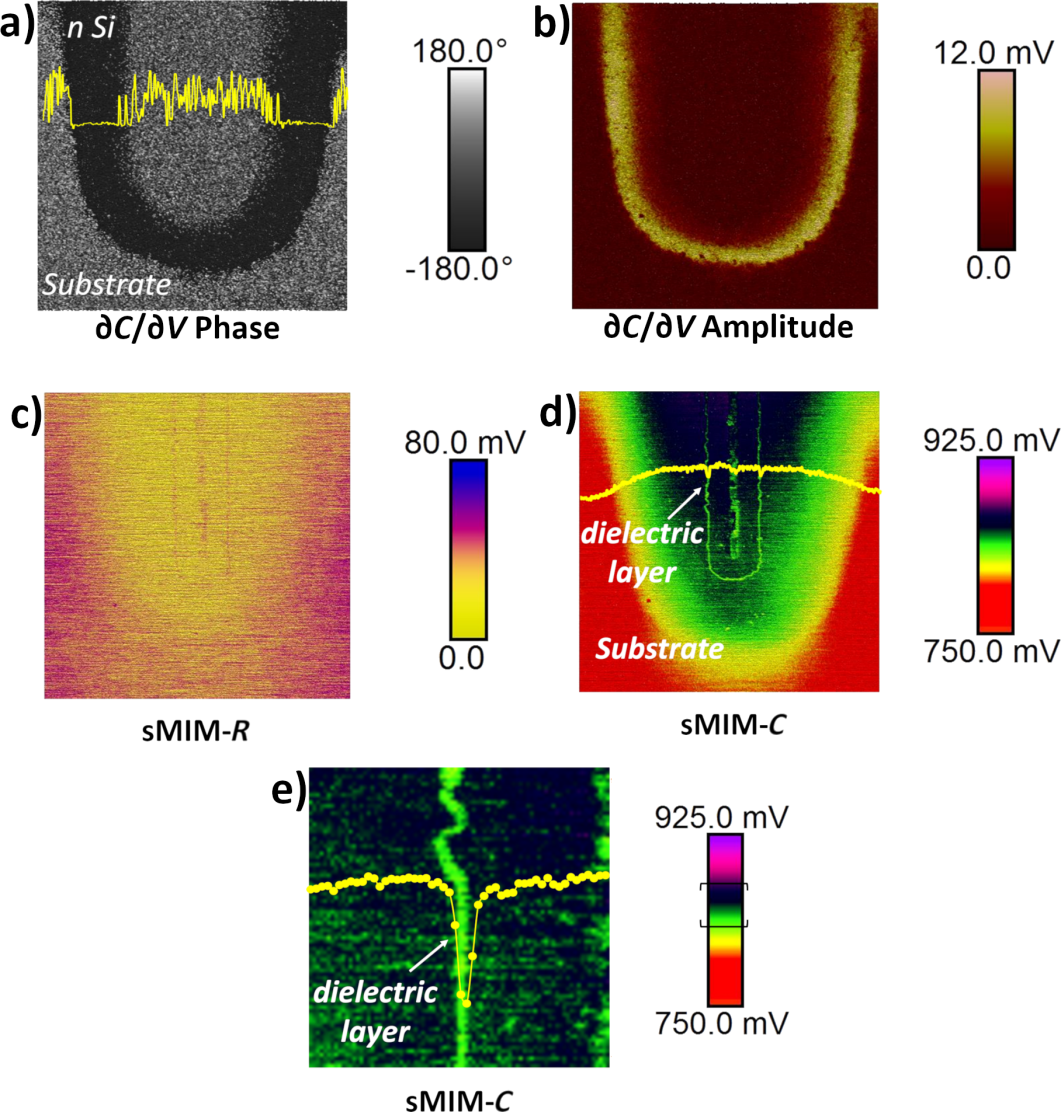
sMIM acquisitions of the bottom of the deep trench structure. (a) ∂*C*/∂*V* phase (10 µm × 10 µm), (b) ∂*C*/∂*V* amplitude, (c) sMIM-*R*, and (d) sMIM-*C* data. The corresponding profiles are represented. (e) A zoomed region (1.5 µm × 1.5 µm) of the dielectric layer shows the high contrast of the sMIM-*C* acquisition, the brackets in the colour-coded scale indicate the data thresholds used.

The second area of interest was the bottom of a deep trench (area 2 in Figure 2). Deep trenches were fabricated by dry etching pores with a high aspect ratio and a diameter of 1 µm. During the FEOL processing, trenches were filled with a dielectric layer, followed by an in situ highly phosphorus-doped polysilicon layer, both deposited by low-pressure chemical vapour deposition (LPCVD). Figure 6 shows the mappings obtained on the cross section of the bottom of a deep trench, showing the ∂*C*/∂*V* phase, ∂*C*/∂*V* amplitude, sMIM-*R*, and sMIM-*C* responses. The signature of the ∂*C*/∂*V* phase (Figure 6a) clearly reveals the n-type character of the implanted and diffused dopants around the deep trench structure into the lightly doped silicon substrate. Furthermore, with the amplitude of the derivative of the capacitance (Figure 6b), information about the filling of the trench (dielectric and doped polysilicon), and again the signature of the diffused doping is provided. At the same time, the sMIM-*C* response (Figure 6d) localizes and reveals, with a very high resolution, the filling oxide and the carrier profile around the deep trench. Even the dielectric layer thickness of 40 nm can be determined (Figure 6d) in the sMIM-*C* results.

**Figure 7:**
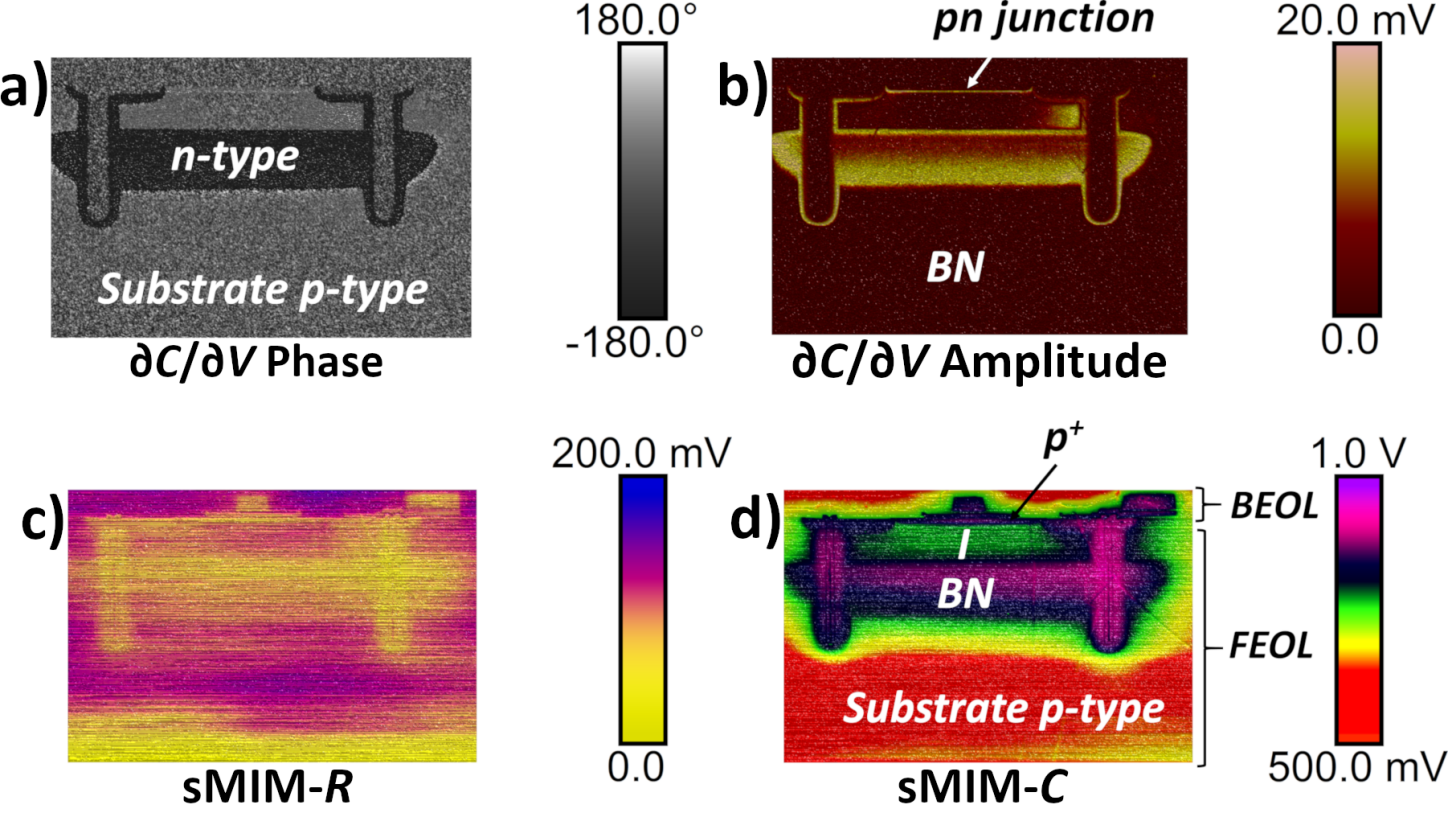
sMIM maps acquired at *V*_DC_ = 0 V on a large area (83 µm × 54 µm) of the PIN diode device. (a) ∂*C*/∂*V* phase, (b) ∂*C*/∂*V* amplitude, (c) sMIM-*R*, and (d) sMIM-*C*. The distinct locations of the different layers of the device are also indicated.

To visualise the global structure of the circular highly integrated PIN diode, the obtained mappings for *V*_DC_ = 0 V on a large scanning area of 83 µm × 54 µm, corresponding to the same area as in Figure 2, are represented in Figure 7. Here, the ∂*C*/∂*V* phase mapping (Figure 7a) highlights the type of doping in different Si layers, such as the BN implanted layer and the diffused doping along the deep trenches. In addition, the ∂*C*/∂*V* amplitude mapping (Figure 7b) identifies the implanted p^+^ anode area and reveals the diffusion shape of the BN implanted layer. sMIM-*R* and sMIM-*C* mappings are shown in Figure 7c and Figure 7d, respectively. Complementary information from the FEOL layers and details of the BEOL layers (metallization, trench dielectric, and isolation) are obtained. It is important to note that the architecture of the metallic contacts (anode and cathode) is also revealed in detail. For this PIN diode switch, the deep trench structures, designed originally for 3D capacitors with a dielectric surface enhancement, are also used to connect the buried layer below the thick lightly doped epitaxial layer. The diffused doping around the deep trenches, used as a cathode to connect the integrated diode, is very well defined by sMIM. The distance between the deep trench rings of the cathode and the anode is fixed at 9 µm. We can also observe a lateral contrast especially in lightly doped areas. This contrast can be explained by the fact that those lightly doped areas are more sensitive to surface charges (often induced by the sample preparation process) and the quality of the native oxide on the scanned silicon surface.

**Figure 8:**
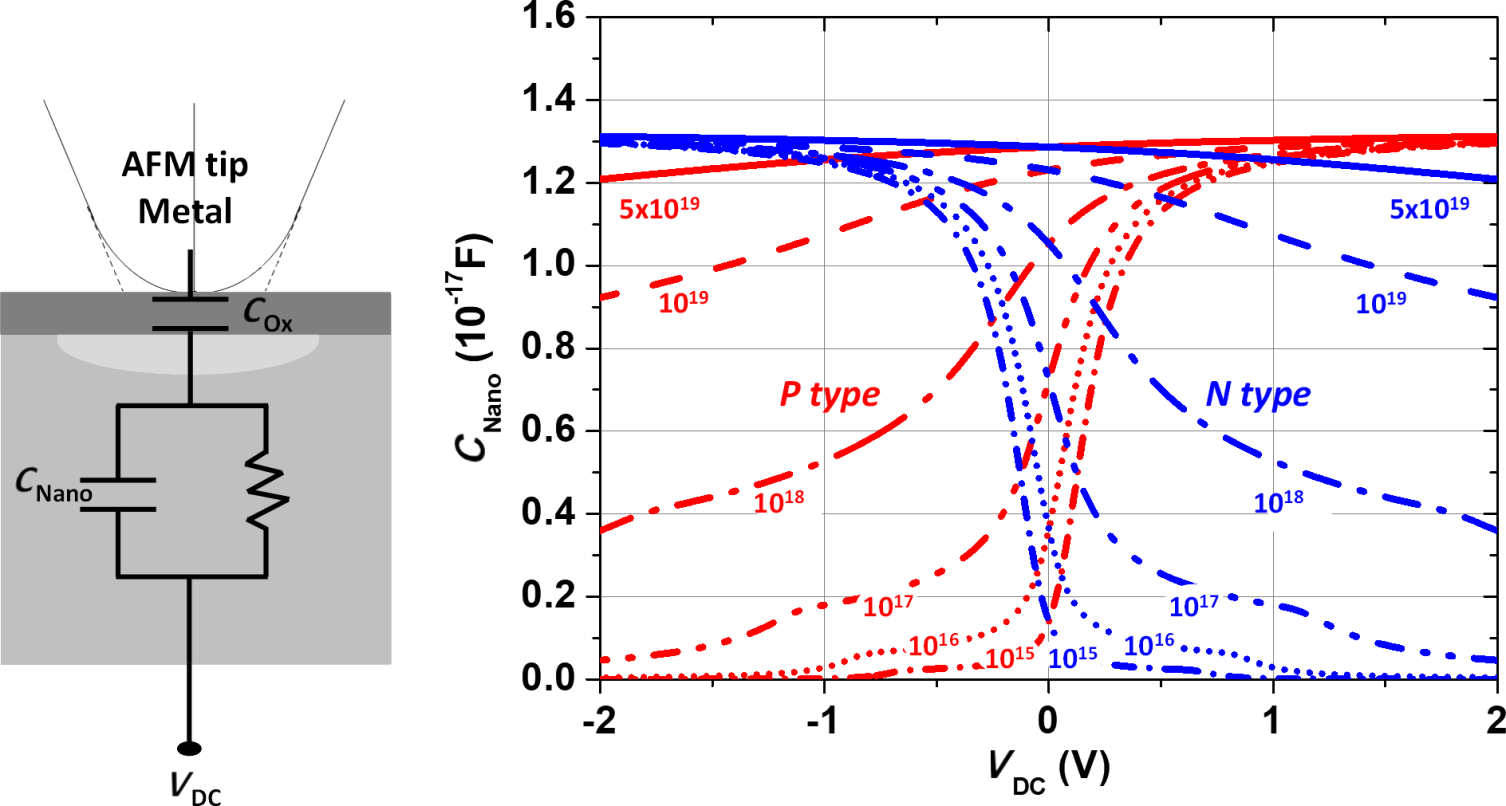
Analytic calculations of the equivalent capacitance *C*_Nano_ determined for the tip–sample contact as a function of *V*_DC_ applied to the sample.

When the sMIM probe scans a doped semiconductor, the tip–sample system forms a nano-MIS capacitor exhibiting a corresponding capacitance *C*_nano_. The sMIM-*C* signal is then proportional to the effective *C*_nano_ of the conductive tip–sample nanoscale contact and varies with the active doping level of the semiconductor. When *V*_AC_ and *V*_DC_ biases are applied, the equivalent nano-MIS capacitor can go either to the accumulation or inversion mode. Figure 8 shows *C*_nano_, determined by our analytic procedure [32], as a function of *V*_DC_ for both n- and p-type semiconductor materials. Using the complete metal–oxide–semiconductor equations, the Poisson–Boltzmann equation is solved for various active dopant concentration values ranging from 10^15^ to 10^19^ atoms·cm^−3^.

**Figure 9:**
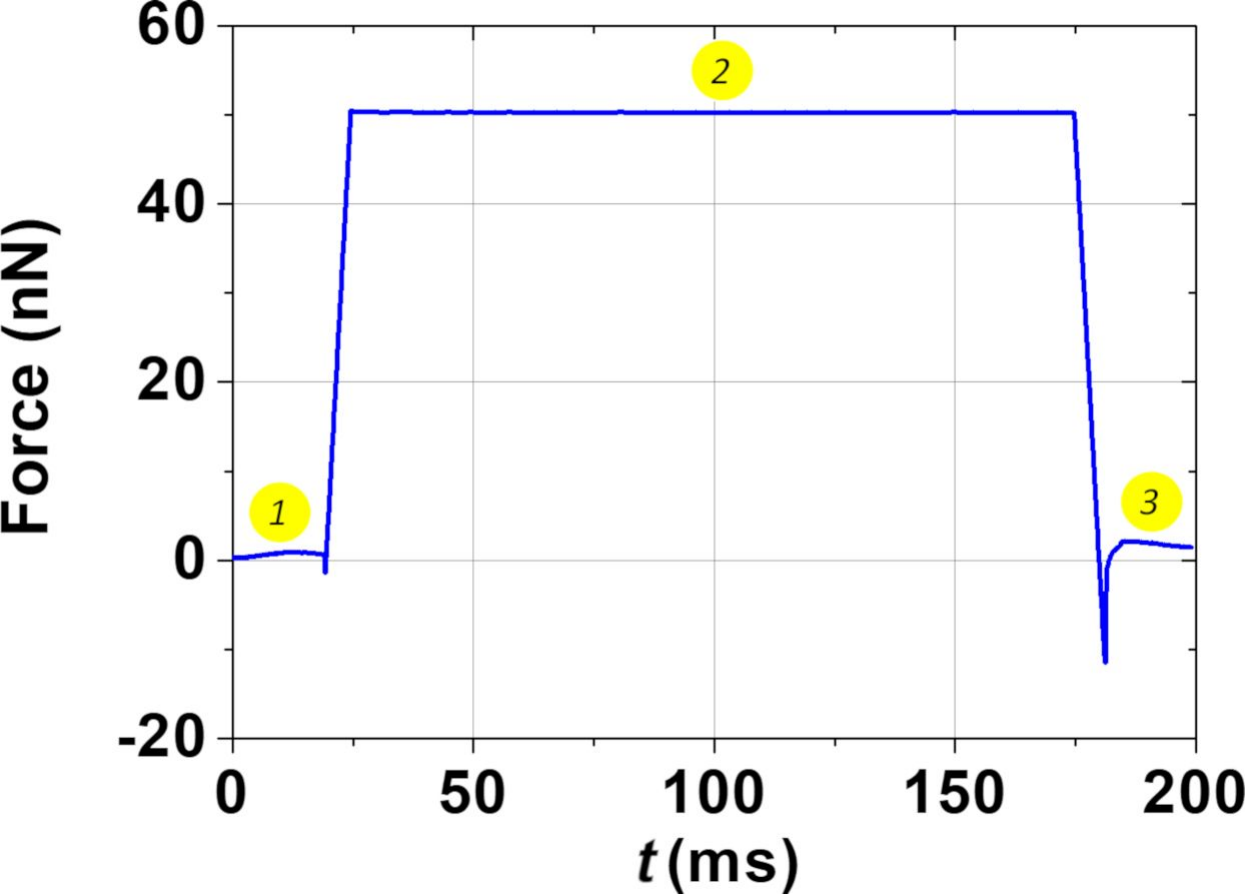
Force curve as a function of time during the dynamic spectroscopy sMIM measurement. 1: tip approach, 2: dwell segment (contact with the surface and multidimensional measurement), 3: tip withdrawal.

In the sMIM spectroscopy mode, a few critical positions on the sample are selected in which the voltage *V*_DC_ is varied while the tip is kept stationary, resulting in electrical *C*–*V* spectra. Therefore, it is possible to complement the imaging analysis by performing dynamic spectroscopy experiments. In our case, we applied a new multidimensional approach [33] (also called DataCube (DCUBE)), which combines sMIM imaging and dynamic sMIM point spectroscopy (at each pixel). This combined mode produces an integrated and complete 3D dataset in which, for every pixel of the mapping, the local variation of the sMIM parameters as a function of the applied bias is recorded. This replaces the subjective approach of guessing the regions of interest (for single-point spectroscopy) by a big data approach, resulting in higher-dimensional data that can be sliced along any axis or plane enabling principal component analysis. Figure 9 represents a force curve recorded as function of time for one pixel. In the first step (1) the tip approaches the surface until the force on the cantilever reaches the setpoint force of 50 nN. During the dwell segment, the sMIM tip is maintained in contact (2). During this time, the voltage sweep is applied in order to record the sMIM measurements as a function of the voltage. Afterwards, the tip is withdrawn from the surface (3).

Therefore, by using the multidimensional approach in the DCUBE analysis, electrical properties over a wide range of bias voltages are measured and images at any applied voltage can be extracted. The availability of a *C*–*V* (sMIM-*C* as a function of *V*) spectrum in every pixel allows for the extraction of a high-resolution map of the flatband voltage and pn junction depletion zones. This illustrates the capability to reveal sample properties that are either not accessible or easily missed in conventional sMIM, in which images are acquired only at one or at a few discrete voltage values.

**Figure 10:**
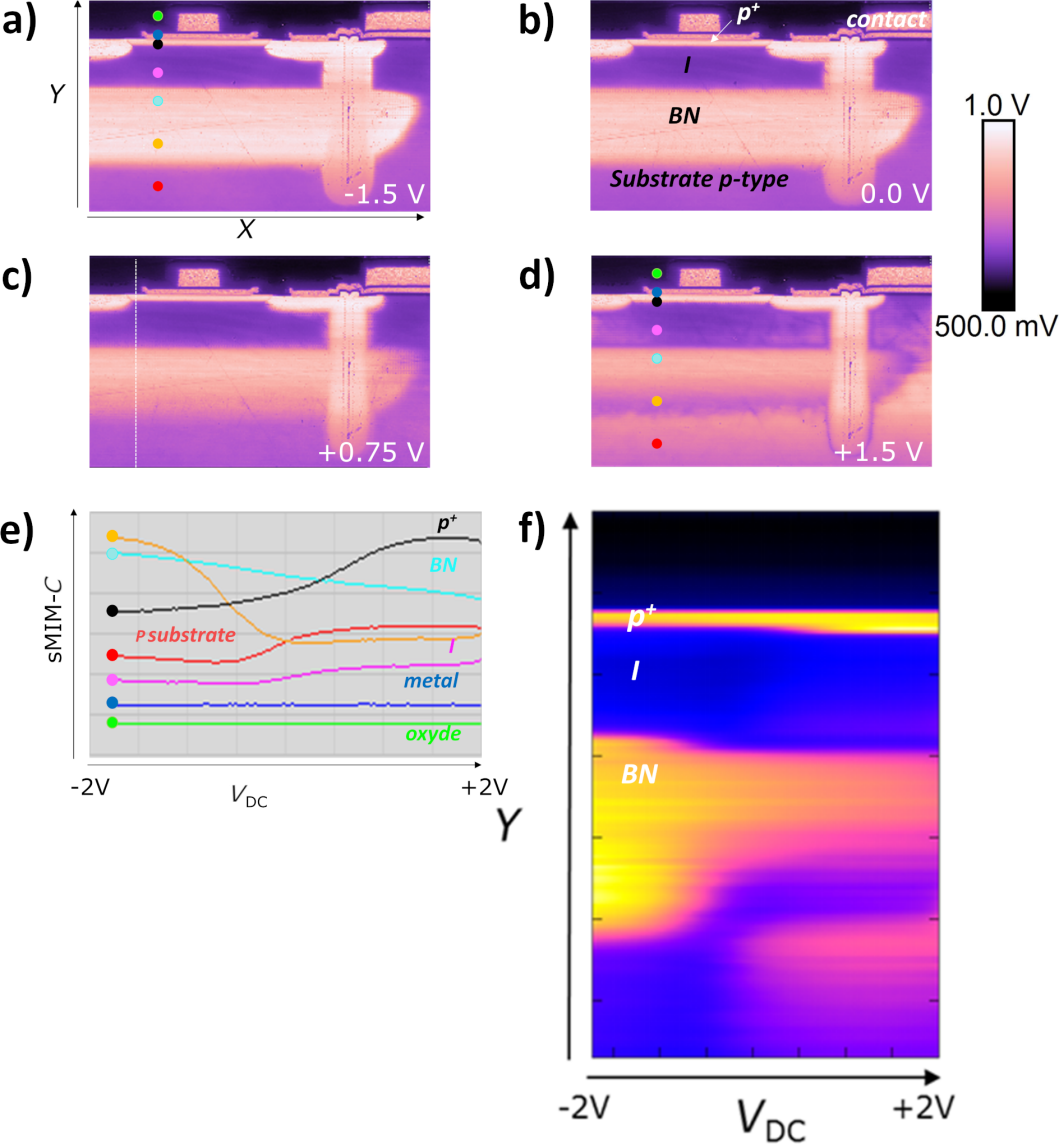
sMIM-*C* DCUBE slices (55 µm × 33 µm) obtained for various *V*_DC_ values (the bias is applied to the sample): (a) −1.5 V, (b) 0 V, (c) +0.75 V, and (d) +1.5 V. (e) The *C*–*V* spectra in selected pixels (−2.0 V to +2.0 V ramp), as indicated by the coloured dots in (a). (f) A spatio-spectral slice showing the *C*–*V* behaviour in every pixel along a selected depth profile, as indicated by the dashed line in (c).

In our analysis, we applied the multidimensional sMIM method on the PIN diode device by sweeping *V*_DC_ from −2.0 V to 2.0 V within 140 ms in every pixel of the image in area 3 of Figure 2. Figure 10 shows typical data extracted from the multidimensional DCUBE sMIM study. The images shown in Figure 10a are the slices obtained during the multidimensional analysis, taken at specific values for *V*_DC_ as indicated in the maps. The images taken in this bias range facilitate the differentiation and localization of all carrier levels and regions in the sample. A positive bias applied to the sample drives the n-type regions into the depletion regime enhancing their contrast, while the p-type regions are in the inversion regime lowering their contrast. When a negative bias is applied, the opposite effect is observed (see Figure 8). Figure 10b shows a few *C*–*V* spectra recorded at selected positions in the PIN diode device (indicated by coloured dots in Figure 10a). We can clearly observe the typical *C*–*V* curve shapes for n-type (cyan dot) and p-type (black dot) regions as well as a flat response in the metal (green dot) and dielectric regions (blue dot). Furthermore, Figure 10c combines this information in a single spatio-spectral slice in which the sMIM-*C* signal is shown as a function of the applied voltage along a selected depth profile, as indicated by the dashed line in Figure 10a. These spatio-spectral plots clearly show how the *C*–*V* spectrum varies along the depth profile, and also how junctions measured in the sMIM images might change under the applied bias. In the BN region, according to analytic calculations (Figure 8), the n-type region increases when *V*_DC_ is decreased. We can also appreciate the evolution of the implanted and diffused n-type doping around the deep trench when the applied voltage varies between −1.5 V and 1.5 V. Applied to a highly integrated device, these results show that the multidimensional sMIM method enables comprehensive studies of the entire architecture of the device including all FEOL and BEOL layers.

In the previous sections, we described the testing methodology of the sMIM measurement for a highly integrated microelectronic device. Both FEOL and BEOL integrated layers were measured and analysed. In our study, sMIM was used to simultaneously record four electrical signals on the nanoscale (∂*C*/∂*V* phase, ∂*C*/∂*V* amplitude, sMIM-*R*, and sMIM-*C*) and the topography. Therefore, we demonstrated that sMIM measurements enable the mapping of local electrical properties of the layers at the nanoscale with very high spatial precision and signal-to-noise ratio. Once the images were obtained, a detailed analysis was necessary to determine the origins of the image contrast. Although the recorded ∂*C*/∂*V* phase clearly indicates the type of the majority carriers detected under the tip contact, the interpretation of the ∂*C*/∂*V* amplitude is more difficult. In fact, as expected, the amplitude of the measured local capacitance variation (∂*C*/∂*V* amplitude) increases when the doping level decreases. This behaviour allows for the localization of the P/N and I/N semiconductor junctions. As a result, the ∂*C*/∂*V* data allows for the discrimination among the dopant types and reveals both homo- and heterojunctions in FEOL layers. However, the very low signal recorded at the location of the quasi-intrinsic layer (I layer) does not enable one to distinguish this lightly doped layer since the recorded signal is close to zero, at the same level of the signal recorded on BEOL layers. The difficulty of detecting changes in the capacitance when only a few carriers are present under the tip–sample nanoscale contact highlights the limitation of the ∂*C*/∂*V* data signal regarding very lightly doped layers.

The simultaneously recorded maps of measured microwave nano-MIS sMIM impedance values evidence the structure of the device. With the sMIM-*R* map, only metallic materials can be distinguished from the semiconductor layers. The low signal-to-noise ratio of the sMIM-*R* signal is related to the fact that sMIM-*R* can be dominated by the effects at the sample surface (including native oxides and surface charge effects). However, the sMIM-*C* results show a very good signal contrast for both p-type and n-type semiconductors at different levels as well as for lightly doped layers, metallic contacts, oxides, and thin dielectric layers. Therefore, the sMIM methodology provides a comprehensive analysis of the overall architecture of the device for both FEOL and BEOL layers. It is important to emphasize that this result shows that, in complement to other AFM-based nanoscale electrical modes and to the local dopant density information, data regarding the BEOL layers is also accessible. Thereby, sMIM can be considered very useful for the failure analysis of several mechanisms, such as interface delineation, local diffusion, or conformity of filling layers. This methodology extends the possibilities to investigate the nanoscale electrical properties and can also solve issues regarding the local interaction between BEOL and FEOL layers (e.g., local diffusion). In addition, while the application of a DC voltage to the sample is not necessary for the measurement of the microwave impedance of the tip–sample system in sMIM, we evaluated the impact of the applied DC voltage using a multidimensional approach that combines imaging and point spectroscopy. This higher-dimensional data provides a comprehensive big data approach regarding the electrical behaviour of the nano-MIS tip–sample system over the whole DC voltage range. Obtained with a single multidimensional scan, sMIM-*C* slices extracted for different DC voltage values (Figure 10) reveal the evolution of the junction delineation as a function of the DC voltage. This new methodology opens many possibilities in the use of nanoscale electrical approaches to understand not only the global device architecture at a single applied DC voltage, but also on a quasicontinuous polarisation spectrum.

## Conclusion

The nanoscale electrical testing methodology of sMIM measurements is presented and applied to an integrated PIN diode. This investigation addresses the complementarity between the ∂*C*/∂*V* and sMIM signals for the characterization of doped semiconductor layers as well as metals and dielectrics. The results and analyses shown highlight the differences and the complementarities of both signals. From the acquisition of the capacitance variation (∂C/∂V) and sMIM signals of the tip–sample nano-MIS, we could identify and localize the electrical properties of doped semiconductors layers from FEOL processing as well as details of the BEOL layers of the processed device. By varying the applied voltage in every pixel of the image, the multidimensional sMIM method (dynamic spectroscopy mode in DataCube) is applied and provides a more complete electrical dataset for each layer of the studied device, with slices of sMIM maps over a wide range of applied voltage values, as well as sMIM-*C*(*V*) spectra in each pixel.

## Supporting Information

This supporting information file presents ∂*C*/∂*V* phase and sMIM-*C* results and analyses obtained for a staircase silicon sample.

File 1 Additional results of scanning microwave impedance microscopy and dynamic spectroscopy-based mapping of integrated PIN diodes.
