# Supplementary material for: Mapping of integrated PIN diodes with a 3D architecture by scanning microwave impedance microscopy and dynamic spectroscopy
Source: Beilstein J Nanotechnol. 2020 Nov 23;11:1764–75. doi: 10.3762/bjnano.11.159 (PMC7705862; doi:10.3762/bjnano.11.159)
Supplement: File 1 — Additional results of scanning microwave impedance microscopy and dynamic spectroscopy-based mapping of integrated PIN diodes. [file Beilstein_J_Nanotechnol-11-1764-s001.pdf]

BEILSTEIN JOURNAL OF NANOTECHNOLOGY

Supporting Information

for

Mapping of integrated PIN diodes with a 3D architecture by scanning microwave impedance microscopy and dynamic spectroscopy

Rosine Coq Germanicus, Peter De Wolf, Florent Lallemand, Catherine Bunel, Serge Bardy, Hugues Murray and Ulrike Lüders

Beilstein J. Nanotechnol. **2020,** *11,* 1764–1775. doi:10.3762/bjnano.11.159

Additional results of scanning microwave impedance microscopy and dynamic spectroscopy-based mapping of integrated PIN diodes

License and Terms: This is a supporting information file under the terms of the Creative Commons Attribution License (https://creativecommons.org/licenses/by/4.0). Please note that the reuse, redistribution and reproduction in particular requires that the authors and source are credited.
The license is subject to the *Beilstein Journal of Nanotechnology* terms and conditions: (https://www.beilstein-journals.org/bjnano)

In this section, we present the results of ∂C/∂V phase and sMIM-*C* obtained for a staircase silicon sample. The sample is an n-type and p-type doping staircase with a carrier profile [1]. The ∂C/∂V image (Figure S1a) shows two levels with a 180° difference between them: the n-type region (yellow area) and the p-type region (dark area). Both n-type and p-type regions consists of a series of stripes, each one of them with a different carrier level, with the highest levels towards the pn junction (i.e., the centre of the image). The monotonic relationship between the sMIM-*C* signal (Figure S1b) and the carrier concentration in the sample is observed for both carrier types. This type of sample can be used for calibrating the sMIM data. Figure 2c shows the *C*–*V* spectra that were collected on each step of the staircase carrier profile. The curves show the expected behaviour, which was also shown in the analytical calculations displayed in Figure 7 of the main manuscript.

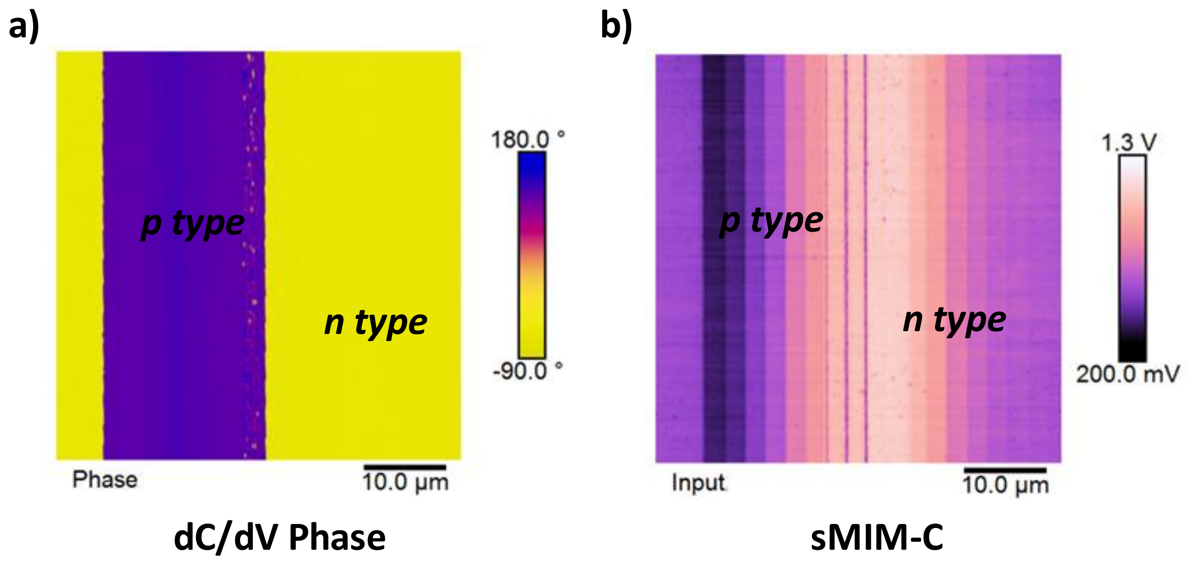

Figure S1: (a) ∂*C*/∂*V* phase and (b) sMIM-*C* maps acquired at V_{DC} = 0 V on the silicon staircase sample.

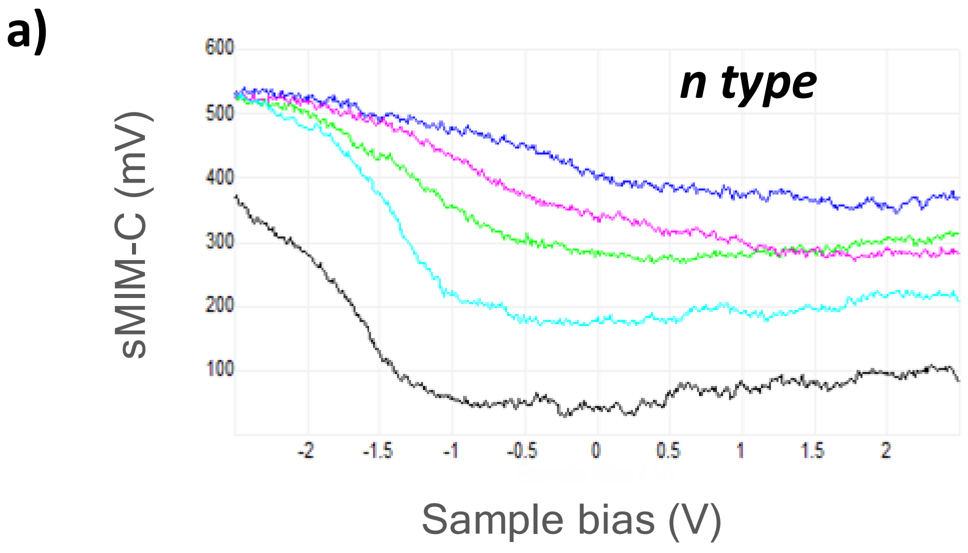

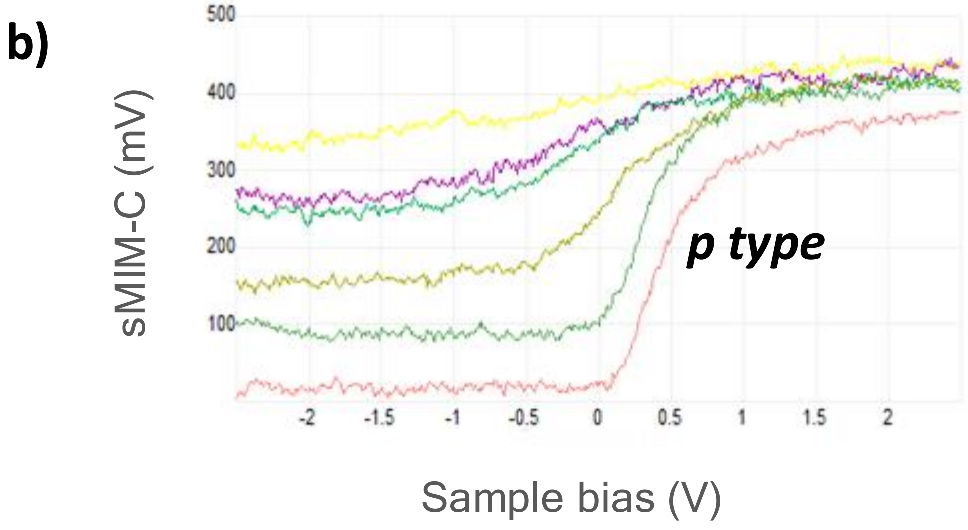

Figure S2: sMIM*C–V* spectra collected with sMIM measurements in (a) the n-type doped regions and (b) p-type doped regions.

References

(1) Wolf, P. D.; Huang, Z.; Pittenger, B.; Dujardin, A.; Febvre, M.; Mariolle, D.; Chevalier, N.; Mueller, T. *Microsc. Today* **2018**, *26* (6), 18–27. doi:10.1017/S1551929518001025
